# Prevalence of group B Streptococcal colonization among pregnant women and neonates in a tertiary hospital in India

**DOI:** 10.4274/jtgga.2017.0032

**Published:** 2017-12-15

**Authors:** Sridhar Santhanam, Ruby Jose, Rani Diana Sahni, Niranjan Thomas, Manisha Madhai Beck

**Affiliations:** 1 Department of Neonatology, Christian Medical College, Vellore, India; 2 Department of Obstetrics and Gynecology, Christian Medical College, Vellore, India; 3 Department of Clinical Microbiology, Christian Medical College, Vellore, India

**Keywords:** Pregnancy, newborn, India, group B Streptococcus, infection

## Abstract

**Objective:**

To estimate the prevalence of group B Streptococcus (GBS) carriage among pregnant women attending the antenatal clinic, and the colonization rates among newborn born to colonized mothers.

**Material and Methods:**

Women attending the antenatal clinic between 35-37 weeks were screened using rectal and lower vaginal swab. Swabs were initially plated on sheep blood agar and LIM broth. The LIM broth was subcultured after 24 hours onto blood agar and CHROMagar StrepB plates with all plates checked for growth at 24 and 48 hours. All babies born to mothers in the study had surface swabs taken to estimate the vertical transmission rate.

**Results:**

Between September 2012 and March 2013, 305 consecutive mothers were screened. Of these, eight mothers were GBS positive in 5% blood agar (2.6%) and 23 mothers showed GBS positivity in enriched media (7.6%). Sixteen of 238 babies (6.7%) were colonized.

**Conclusion:**

Though lower than rates from most countries, 7.6% of mothers attending an antenatal clinic in south India were colonized with GBS. Use of enrichment media markedly increased the detection rate. Approximately two-thirds of newborn born to colonized mothers were also colonized. There were no instances of invasive GBS disease, indirectly proving the efficacy of intrapartum prophylaxis in preventing neonatal GBS disease.

## INTRODUCTION

Group B Streptococcus (GBS) is one of the major causes of perinatal infections. It causes sepsis, meningitis, and pneumonia in the newborn and young infants. In the mother, it is one of the important causes of chorioamnionitis, post-partum endometritis, urinary tract infections, post cesarean febrile illness, and rarely, endocarditis..

GBS colonizes the lower genito-urinary and gastro intestinal tracts in adults, with the colonization being chronic or intermittent. In the United States of America (USA), it is estimated that 15-40% of pregnant women are carriers of GBS (1). In a colonized woman, the bacteria can be transmitted to the fetus in the intra-uterine or perinatal period. This transmission from the mother to the newborn occurs variably and the transmission rate is estimated to be between 40-73%. Of the babies born to colonized mothers, 1-2% develop infection in the immediate neonatal period (early onset sepsis).

Reduction of this vertical transmission of GBS to the newborn has been a priority over the past three decades. The method that has proved most successful has been screening of all pregnant women during pregnancy, with intrapartum antibiotics given to colonized women in labor. Using this strategy, GBS infection among newborn in the USA has been reduced from 1.7-1.9 per 1000 live births in the early 1990s, to 0.34-0.37 per 1000 newborn in 2008 (2). Cost-benefit analysis shows that this strategy would be most helpful in regions where neonatal GBS sepsis prevalence is high (more than 1.2 per 1000 live births). In regions where the prevalence of GBS infection in newborn is low, intra partum antibiotics given in the presence of specific maternal risk factors deemed to be high risk for transmission to the fetus/newborn might be more useful.

GBS carriage in mothers varies between geographic regions and races. Studies in various developed nations have shown GBS carriage rates of between 20-40%. The few studies published from developing countries have shown comparatively lower prevalence rates. There have been only two studies from the Indian subcontinent on GBS carriage among pregnant women. One study from our institution in the early 1980s showed a prevalence of 5.8% (3). The other study from Pondicherry in south India showed a prevalence of 2.3% (4).

The prevalence of neonatal early onset sepsis with GBS in our hospital was 0.17/1000 live births in the period 1988-1997 (5). This has increased subsequently to 0.68/1000 live births between 1998 and 2010 (6). We hypothesized that maternal carriage rates must have increased in these two decades to cause the increase in newborn early-onset infections; hence the need to document maternal carriage rates among pregnant women. This would help to decide whether universal screening should be offered during pregnancy to prevent maternal and neonatal morbidity.

## MATERIAL AND METHODS

The study was conducted in the Obstetric and Neonatology departments of a tertiary level perinatal hospital in south India that has around 13.000 deliveries/year. All pregnant women booked with Obstetric Unit IV were approached for screening and those who consented to take part and were likely to deliver in this hospital were recruited.

Swabs were taken from the lower vagina and rectum as per the Centers for Disease Control and Prevention guidelines (2).The swabs were transported immediately to the Clinical Microbiology Department where they were plated directly on quality passed 5% sheep blood agar plates prepared in-house, CHROMagar StrepB (CHROMagar, France) and inoculated in Lim broth (Becton, Dickinson and Co., NJ). Following incubation for 24 hours at 37 °C under 5% CO_2_ atmospheric air, the Lim broth was then subcultured onto both blood agar and CHROMagar StrepB plates. The primary plates were checked for growth at 24 and 48 hours. The plates subcultured from Lim broth were also checked for growth at 24 and 48 hours. Plates were then classified as showing no growth or growth of GBS. When growth occurred in the primary culture itself, it was deemed to be heavy colonization, and if it occurred only in Lim broth, it was light colonization (7).

GBS was identified by either beta or gamma hemolysis on blood agar, mauve colonies on chromogenic media, and a positive latex agglutination with group B antisera (Plasmatec laboratories Ltd, UK). Organism identification was confirmed using the Christie, Atkins, and Munch-Petersen test. Beta-hemolytic ATCC 12386 Streptococcus agalactiae and non-hemolytic ATCC 13813 S. agalactiae were used as quality control strains. Mothers who showed positive growth for GBS received intra partum antibiotic prophylaxis with ampicillin.

All babies born to mothers who participated in the study had surface swabs (umbilical and external ear) taken within 30 minutes of delivery to estimate the newborn colonization rates. Babies born to mothers who were colonized as well as those with traditional risk factors for early-onset sepsis had a peripheral blood cultures taken (irrespective of maternal intrapartum antibiotic status) and were started on antibiotics (crystalline penicillin and gentamicin) until the culture showed no growth.

Details of the mother’s medical and obstetric history, along with delivery details were recorded. The baby’s birth demographics and neonatal course was also documented.

**Sample size:** The study conducted in our hospital in 1982 showed a prevalence of 5.8%. Hence, the estimated sample size for a prevalence of 6% with a precision of 3% needed a minimum of 241 patients to be screened. Allowing for 20% dropout, we planned to screen 290 mothers.

Analyses were performed using SPSS 13.5. Prevalence rates were calculated as percentages.

The study was approved by the institutional review board. The study complied with the ethical conduct of research using human subjects.

## RESULTS

A total of 305 pregnant women were screened between September 2012 and March 2013. The mean age of the women in the cohort was 26±4.2 years. Most women were in the 26-30-years’ age range (33.4%) and a large proportion of them (62%) were primiparous.

The primary cultures were positive for GBS in eight mothers (2.6%) (heavy colonization), and 23 swabs were positive in the Lim broth subcultures (7.64%) (light colonization). Two hundred thirty-six of the 305 mothers delivered 238 babies in this hospital and the babies were evaluated with surface swabs and sepsis screen. This included 20 of the women who were GBS-positive. The other women delivered elsewhere and hence the babies could not be screened. Of the women who were GBS-positive, 6 did not receive intrapartum antibiotics. The surface swabs from 3 newborn babies were positive for GBS on primary cultures (1.3%), and 16 after inoculation in enriched media (6.7%). The latter included three babies whose mothers were not found to be colonized. None of the babies were symptomatic or had positive blood culture for GBS.

## DISCUSSION

GBS has been identified as an important cause of infection in the perinatal period in both the mother and her newborn. This bacterium is of particular interest because of the fact that intrapartum antibiotics given to colonized mothers can reduce the burden of early-onset disease in the newborn. This requires screening of pregnant women late in pregnancy or in labor and administration of antibiotics to those colonized. The prevalence of colonization varies with geographic region, sociodemographic status, ethnicity, and sexual activity. In low prevalence areas, it might be more cost effective to give intrapartum antibiotics to mothers in labor with certain identified risk factors that place their newborn at higher risk of early-onset infection.

Studies in various developed nations have shown different GBS carriage rates: Canada 19.5% (8), the United Kingdom (Oxford) 21.3% (9), the USA 15-40% (1), and Sweden 25.3% (10). The few studies published from developing countries have shown comparatively lower prevalence rates: Lebanon 17.7% (11), Brazil 17.9% (12), India (Vellore) 5.8% (3), and Pondicherry 2.3% (4). The exception is Zimbabwe, where colonization rates of 60.3% were noted (13).

The incidence of early-onset infection in the newborn has increased four-fold in the last two decades in our hospital compared with the 1990s, inspite of risk-based intrapartum antibiotics being given to mothers in labor since 2004. In the present study, we found that the colonization rate was 7.6%, which is an increase from the previous study, and also much higher than the study from Pondicherry. It is, however, much less than the prevalence rates quoted in studies from other low- and middle-income countries. Thus, it might be argued that GBS colonization rates are lower in India compared with most high- or middle-income countries.

Our study showed that nearly 65% of newborn born to GBS-positive women were colonized with GBS, though none developed invasive infection. This might point to the effectiveness of intrapartum antibiotics in preventing invasive disease. Thus, it was seen that the GBS colonization rate had increased in pregnant women since the 1980s, but was still far below the prevalence rates seen in developed countries and other middle-to-low income countries. It is also not commensurate with the multi-fold increase in early-onset neonatal sepsis. Whether screening in labor is therefore the next logical solution is to be determined.

The transmission rate to the fetus was high, though there were no cases of invasive disease in the newborn. Therefore, it appears that universal maternal screening for GBS may, at present, be cost-ineffective in India. Selective screening is not possible because there are no significant risk factors identifiable for maternal colonization. Risk-based antibiotic prophylaxis to mothers and selective sepsis examinations in newborn would be the best choice in this scenario to prevent early-onset GBS disease in newborn.
